# Potential use of the alkaline comet assay as a predictor of bladder tumour response to radiation

**DOI:** 10.1038/sj.bjc.6601426

**Published:** 2003-12-09

**Authors:** S R McKeown, T Robson, M E Price, E T S Ho, D G Hirst, V J McKelvey-Martin

**Affiliations:** 1 Radiation Science Research Group, University of Ulster, Jordanstown, Northern Ireland; 2 Urology Department, Belfast City Hospital, Belfast, Northern Ireland; 3 Cancer and Ageing Research Group, University of Ulster, Coleraine, Northern Ireland

**Keywords:** bladder cancer, comet assay, radiosensitivity, predictive test

## Abstract

Bladder tumours show a variable response to radiotherapy with only about 50% showing good local control; currently there is no test to predict outcome prior to treatment. We have used five bladder tumour cell lines (T24, UM-UC-3, TCC-SUP, RT112, HT1376) to investigate the potential of the alkaline comet assay (ACA) to predict radiosensitivity. Radiation-induced DNA damage and repair were compared to clonogenic survival. When the five cell lines were irradiated and initial DNA damage was plotted against cell survival, at all doses (0–6 Gy), a significant correlation was found (*r*^2^=0.9514). Following 4 Gy X-irradiation, all cell lines, except T24, showed a correlation between SF2 *vs* half-time for repair and SF2 *vs* residual damage at 5, 10, 20 and 30 min. The T24 cell line showed radioresistance at low doses (0–2 Gy) and radiosensitivity at higher doses (4–6 Gy) using both cell survival and ACA end points, explaining the lack of correlation observed for this cell line. These data indicate that initial DNA damage and residual damage can be used to predict for radiosensitivity. Our data suggest that predictive tests of radiosensitivity, appropriate to the clinical situation, may require the use of test doses in the clinical range.

West and colleagues have shown that biopsies from cervix and head and neck tumours can be used to predict for an individual patient's response to radiotherapy using clonogenic cell survival following exposure to 2 Gy X-irradiation (West *et al*, 1997; Bjork-Eriksson *et al*, 2000). The main disadvantages of this assay are that a minimum of 4 weeks is required to obtain a result, and about 30% of excised tumours failed to grow in soft agar; success of this method in other tumour types has been elusive. The limitations of this assay have stimulated an interest in developing methods that might provide a quicker, and more reliable, measure of tumour radiosensitivity so that results could be considered in treatment planning; to date no satisfactory test has been reported.

A number of studies have investigated the utility of the neutral comet assay (NCA) as a predictive method for radiosensitivity with varying results. Olive *et al*, (1994) showed no correlation with radiosensitivity for six human tumour cell lines of different origins, whereas a reasonable correlation has been reported in two studies when the cell lines used were of the same tissue of origin, that is, cervix (Marples *et al*, 1998) and bladder (Price *et al*, 2000). However, Woudstra *et al* (1998), using the related methods of pulse field gel electrophoresis (PFGE) and the halo assay, could not show correlation, using 10 human tumour cell lines of different tissue origins. One particular drawback of the NCA, and many of the other DSB assays is that, for reasons of poor sensitivity, they are carried out at doses well outside the clinically relevant range (approximately 20–150 Gy). This makes comparison of the results to clonogenic assays, normally carried out between 0 and 10 Gy problematic.

The alkaline comet assay (ACA) has several attractive features for a predictive test of radiosensitivity: it is simple and quick to do, it does not require clonogenic cell growth as it is carried out on single-cell suspensions from primary tumours and effects can be measured in the clinically relevant dose range (0–6 Gy). All of these criteria are essential if a predictive test is to have practical utility in a clinical context. Although there are many studies in the literature on the value of the ACA in the assessment of radiosensitivity, the majority are on normal cells, for example, lymphocytes or fibroblasts. Investigation of the utility of the ACA for determining tumour cell radiosensitivity has been limited to a few studies, often comparing cells from a range of tumours types (Table 1

**Table 1:** Studies using the ACA to measure DNA damage and repair in tumour cells following exposure to X-irradiation

		**Radiation Dose range/time of repair study**		
**Reference**	**Sample**	**ID^a^**	**Repair**	**Main conclusions**
Olive *et al* (1990)	Mouse tumour cells (^*^SCC VII) and tumour-derived macrophages	0–15 Gy	15 Gy 0–30 min	ID: increased linearly with dose, similar in normal and tumour cells Repair: no difference
Müller *et al* (1994)	Human SCCs (PECA 4451, PECA 4197), human melanoma (MeWo)	0–2 Gy	5 Gy 0–120 min	ID: no correlation Repair: Some correlation
McKelvey-Martin *et al* (1998)	Three human bladder tumour cell lines (RT112, UM-UC-3, HT1376)	0–10 Gy	ND	ID: inverse correlation SF_2_ and tail moment
Bergqvist *et al* (1998)	Four human lung tumour cell lines (U-1285, U-1906E, U-1752, U-1810)	0–5 Gy	0–5 Gy 60 min	ID: No correlation with SF_2_ RD: some correlation at 60 min
Bachova *et al* (2002)	Three human ovarian tumour cell lines (CH-1, A-2780, SKOV-3)	0, 2, 8 Gy	2 and 8 Gy 0–90 min	ID: correlation with apoptosis Repair rate: no correlation RD: some correlation at 30 min

a SCC=squamous cell carcinoma; ID=initial damage; RD=residual damage; ND=not determined.

). Previously, we have reported a preliminary study of three cell lines derived from transitional cell carcinoma (TCC) of the bladder. Cells were exposed to 0–10 Gy X-rays and an inverse correlation between cell survival (clonogenic assay) and mean tail moment (ACA) was observed (McKelvey-Martin *et al*, 1998). This supported the hypothesis that the ACA might be useful in predicting the radioresponsiveness of individual cell lines. Here, we report results in five bladder cancer cell lines using a modified, more sensitive, version of the ACA. Our results are further supported by two independent studies using colorectal tumour cells (Dunne *et al*, 2003) and bladder tumour cells (Moneef *et al*, 2003).

## MATERIALS AND METHODS

### Cell lines and culture

Five mycoplasma-free bladder cell lines, derived from high-grade TCCs were used in this study (UM-UC-3, RT112, HT1376, TCC-SUP and T24; American Tissue Culture Collection). All of the cell lines, except T24, were maintained in exponential growth in Eagle's minimal essential medium (EMEM), supplemented with 2 mM l-glutamine, 25 mM sodium bicarbonate, 10% foetal calf serum (FCS), 1% nonessential aminoacids, 1% penicillin–streptomycin; for UM-UC-3 cells 1% sodium pyruvate was also added. The T24 cell line was grown in McCoy's medium supplemented as above. Doubling times of the cell lines were between 20 h (UM-UC-3) and 36 h (HT1376). Cells were harvested at 80–90% confluence.

### X-ray irradiation for comet assays

Cells were irradiated either on slides or in Eppendorf tubes at 4°C using a Siemans Stabilipan X-ray machine operated at 300 kV at a dose rate of 2.6 Gy min^−1^. Repair studies were performed by irradiating preembedded cells on crushed ice; slides were then placed in ice-cold lysis buffer (control) or in growth medium at 37°C in 95% air: 5% CO_2_ for specified times prior to lysis.

### Alkaline Comet Assay

The Eppendorf method was performed using a protocol developed by Singh *et al* (1988). Briefly, Dakin fully frosted slides were covered with 300 *μ*l of 0.75% normal melting point agarose (NMP), dissolved in PBS at 45°C, coverslips were added and the agarose allowed to solidify. Cells (10^5^) in 1 ml culture medium were irradiated in Eppendorf tubes on ice before centrifugation at 4°C for 5 min at 1200 r.p.m. The coverslips were removed and 89 *μ*l of 0.75% low melting agarose (LMP), dissolved in PBS at 37°C, was added to each pellet (0.11 *μ*l); the final concentration of agarose was 0.67%. The cell/agarose suspension was used to form the second layer and allowed to solidify under a fresh coverslip at room temperature for 5 min. The embedded cells, with coverslips removed, were then placed at 4°C in cold lysis buffer (2.5 M NaCl, 100 mM Na_2_EDTA, 10 mM Tris, pH 10 and 1% Triton X-100 added fresh). After 1 h, slides were drained and placed in a horizontal gel electrophoresis unit containing fresh chilled electrophoresis buffer (300 mM NaOH and 1 mM Na_2_EDTA, pH 13.0) to a level of approximately 0.25 cm above the slides. Slides were kept in this buffer for 20 min to allow unwinding of the DNA. Electrophoresis was carried out for 20 min at 25 V (0.83 V cm^−1^). Slides were drained, placed on a tray and flooded slowly with three changes of neutralisation buffer (0.4 M Tris pH 7.5) each for 5 min. Slides were stained with 50 *μ*l of ethidium bromide (20 *μ*g ml^−1^) and covered with a coverslip for immediate analysis. All the steps were conducted under yellow light to prevent additional DNA damage by natural light. Analysis was carried out on 25 cells/slide, two slides per dose/time point, that is 50 values per point; each experiment was carried out on three separate occasions.

The slide method is a modification of the Eppendorf method to allow irradiation of cells after embedding in low melting point (LMP) agarose. Briefly, 95 *μ*l of 0.6% NMP agarose, in RPMI medium, was allowed to solidify on Dakin slides (as above). Equal volumes of cell suspension (2 × 10^5^ cells ml^−1^ and 1.2% LMP agarose (in RPMI medium containing 10% FCS, at 37°C) were mixed. A volume of 75 *μ*l of this second layer was quickly pipetted on to the NMP agarose-coated slides and allowed to solidify under a coverslip for 5 min on ice. Coverslips were removed and the slides were irradiated (0–6 Gy) on ice. The slides were immediately immersed in ice-cold lysis buffer and the remaining steps were as described above. We confirmed that the difference in LMP agarose concentration for the two methods (0.67 *vs* 0.6%) had no significant effect on tail moment (unpublished data).

Cells were analysed using Hewlett Packard Super VGA and Fenestra Komet Software (version 3) (Kinetic Imaging Ltd.). Observations were made at a magnification × 400 using an epi-fluorescence microscope (Olympus BH2) equipped with an excitation filter of 515–535 nm, 100 W mercury lamp and a barrier filter at 590 nm. Several parameters of each cell were calculated by the software package, and tail moment was selected as the parameter that best reflected DNA damage. Tail moment is defined as tail length multiplied by the percent tail DNA, where tail length is defined as comet length minus head length.

### Clonogenic assay

Single cell suspensions were counted and seeded in appropriate numbers in 60 × 15 mm Petri dishes (Falcon) with 6 ml appropriate culture media. Following a 4 h incubation at 37°C, cells were irradiated at 2, 4, 6, 8 and 10 Gy; unirradiated cells were processed in parallel. Cells were then incubated at 37°C, in a humidified 95% air, 5% CO_2_ for 2 weeks, stained with crystal violet and colonies (50 or more cells) were counted. Three replicates were prepared for each control and dose group, and each experiment was performed on three separate occasions. For each cell line, the plating efficiency (PE) for untreated cells was determined (this was >35% for all cell lines). The surviving fraction (SF) was defined as the ratio of colonies produced to cells plated, with a correction for PE. SF=colonies counted/cells seeded × (PE/100).

### Statistical analysis

Standard errors for individual results were calculated from three means derived from individual replicate experiments. To determine significance, data were subjected to one factor ANOVA (*P*-values <0.05 were considered significant). To derive correlation coefficients, a linear curve fit was applied to the data for all cell lines excluding T24. The data for T24 were omitted as this cell line clearly changes its sensitivity to radiation over the range 2–4 Gy; therefore, repair at 4 Gy cannot be compared to the SF2 which is measured at 2 Gy (see Discussion below).

## RESULTS

**Figure 1:**
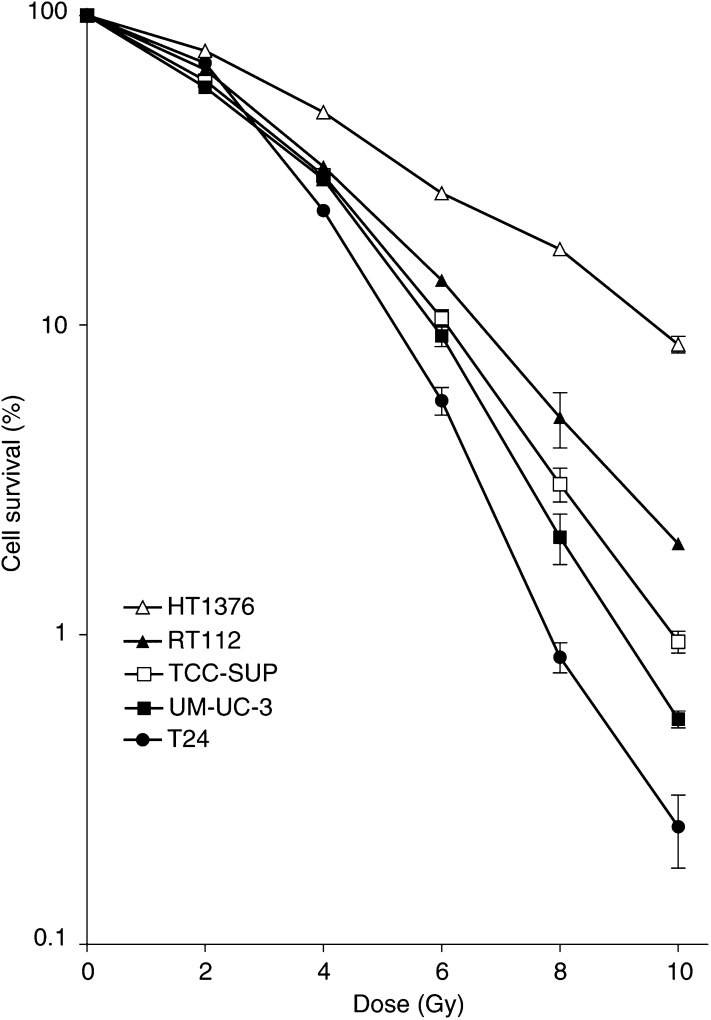
Survival of T24, UM-UC-3, TCC-SUP, RT112 and HT1376 cells. Cell survival was measured as the number of colonies formed following X-ray irradiation at 0–10 Gy. Each data point is the mean±s.e. of three separate experiments. Each experiment contained three replicates. Error bars are shown only when they exceed the size of the symbol.

Radiation survival curves for the five bladder cancer cell lines are shown in Figure 1

, with the surviving fraction at 2 Gy (SF2) in Table 2

**Table 2:** Repair of DNA damage in five bladder cancer cell lines following exposure to X-irradiation

**Cell Line**	**SF2^a^**	**Residual damage (30 min)^b^**	**Residual damage (60 min)^b^**	***T*_1/2_^c^(min)**
T24	0.70±0.01	4.38±0.25	2.31±0.37	8.8
UM-UC-3	0.59±0.03	1.06±0.07	0.34±0.02	5
TCC-SUP	0.62±0.02	0.84±0.01	0.18±0.02	4
RT112	0.67±0.01	0.50±0.03	0.00±0.002	3.0
HT1376	0.77±0.07	0.00±0.002	0.00±0.002	2.5

a Surviving fraction of cells exposured to 2 Gy, determined by performing three independent experiments each containing three replicates.

b Residual DNA damage calculated by subtracting the control tail moment from that remaining at 30 and 60 min in Figure 5.

c Time (min) for half of the initial damage to be repaired. The data represent the mean±s.e. of three independent experiments.

. The cell lines encompass a range of sensitivities with HT1376 being the most radioresistant cell line and UM-UC-3 the most radiosensitive. T24 showed a shallow shoulder on the survival curve leading to a radioresistant response at low doses; however, there was a greater rate of change of radiosensitivity above 2 Gy, which lead a radiosensitive response at higher doses. This result has been confirmed by Moneef *et al* (2003).

**Figure 2:**
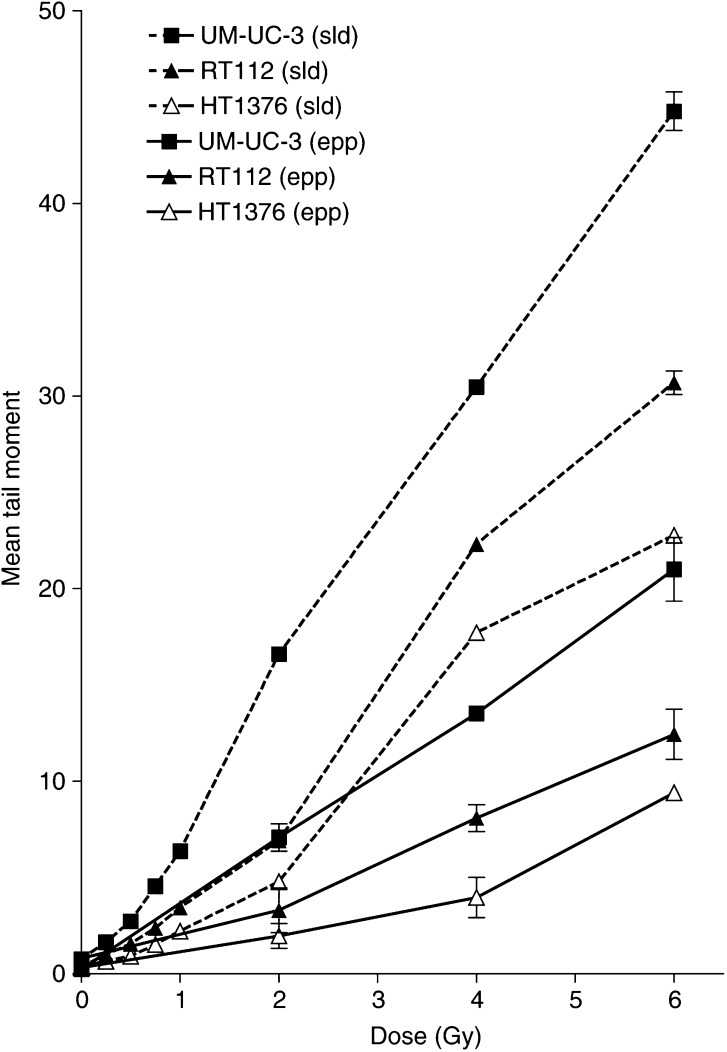
DNA damage in UM-UC-3, RT112 and HT1376 cells using the Eppendorf and slide ACA. Cells were irradiated on ice. DNA damage was measured as tail moment. Data points represent the mean±s.e. from three independent experiments. Error bars are shown only when they exceed the size of the symbol.

DNA damage following irradiation was measured in three cell lines using both the Eppendorf and slide variants of the ACA. Reproducible dose–response curves for mean tail moment were obtained, with the radiosensitive cell line (UM-UC-3) displaying the greatest DNA damage and the radioresistant cell line (HT1376) displaying the least (Figure 2

). The slide version of the comet assay resulted in a 2–3-fold increase in sensitivity for the detection of DNA damage. The level of background damage in unirradiated controls for individual cell lines was similar (tail moment≅0.40). The Eppendorf method gave a significant difference (*P*<0.01, one factor ANOVA) in tail moment between different cell lines for radiation doses of 4 Gy and above; the slide method revealed a significant difference at 1 Gy (*P*<0.05, one factor ANOVA).

**Figure 3:**
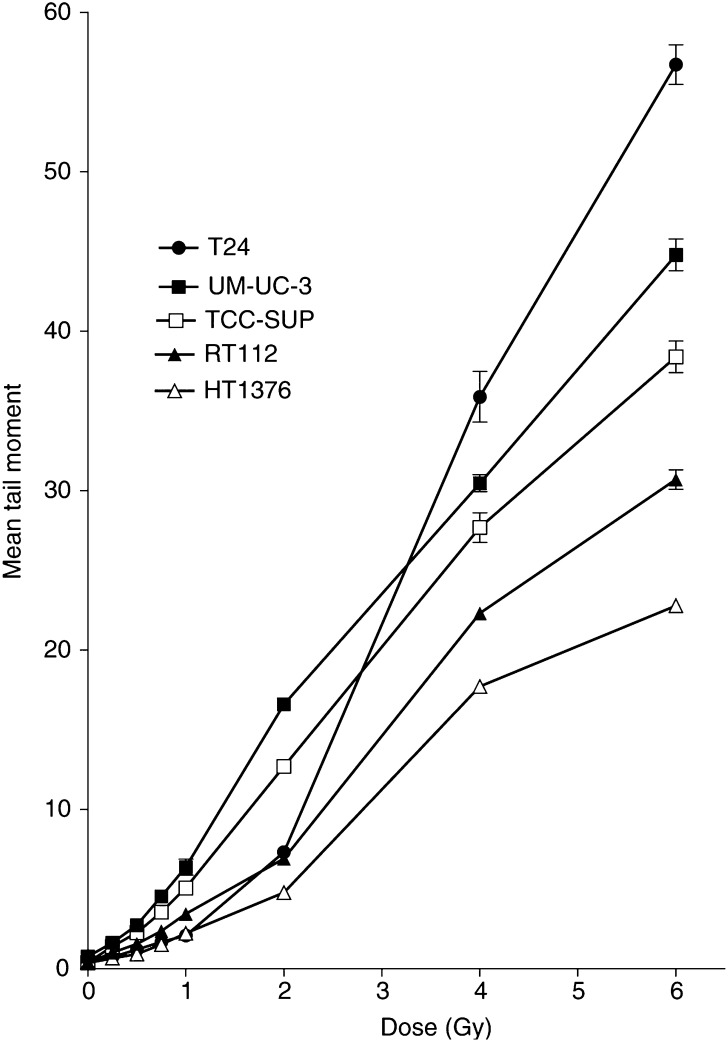
DNA damage in T24, UM-UC-3, TCC- SUP, RT112 and HT1376 cells. DNA damage was measured by mean tail moment using the slide comet assay. Cells were irradiated at 0–6 Gy on ice. Data points represent the mean±s.e. from three independent experiments. Error bars are shown only when they exceed the size of the symbol.

When the five cell lines are compared using the slide comet assay, the radiosensitive lines (e.g. UM-UC-3) showed the greatest DNA damage and the radioresistant lines (e.g. HT1376) displayed the least damage (Figure 3

). Figure 4

**Figure 4:**
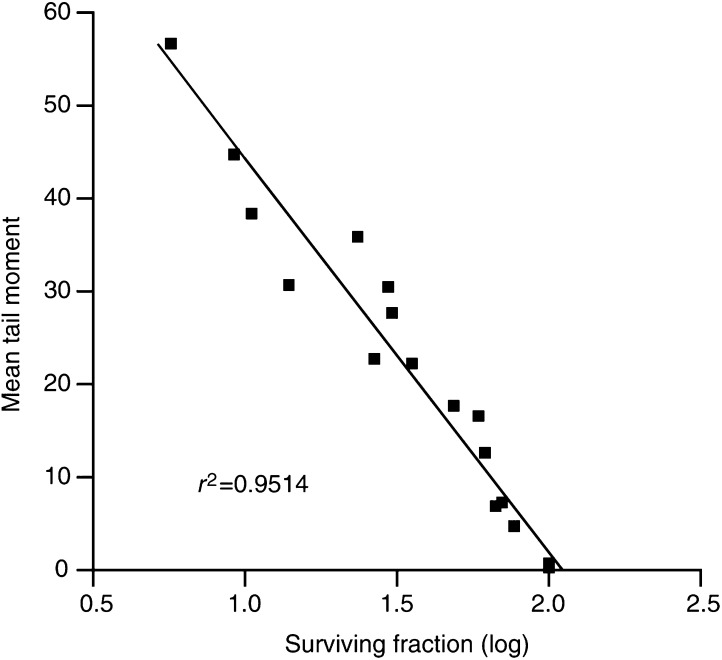
Correlation between cell survival and mean tail moment following exposure to 0–6 Gy irradiation in T24, UM-UC-3, TCC-SUP, RT112 and HT1376 cells. Cell survival was determined using a clonogenic assay, tail moment was measured using the comet assay (slide method).

shows mean tail moment for initial damage (0–6 Gy) plotted against log cell survival (data from all five cell lines) and a significant correlation is found (*r*^2^=0.9514). This suggests that, at low clinically relevant doses, the ACA could be used to predict cell survival.

**Figure 5:**
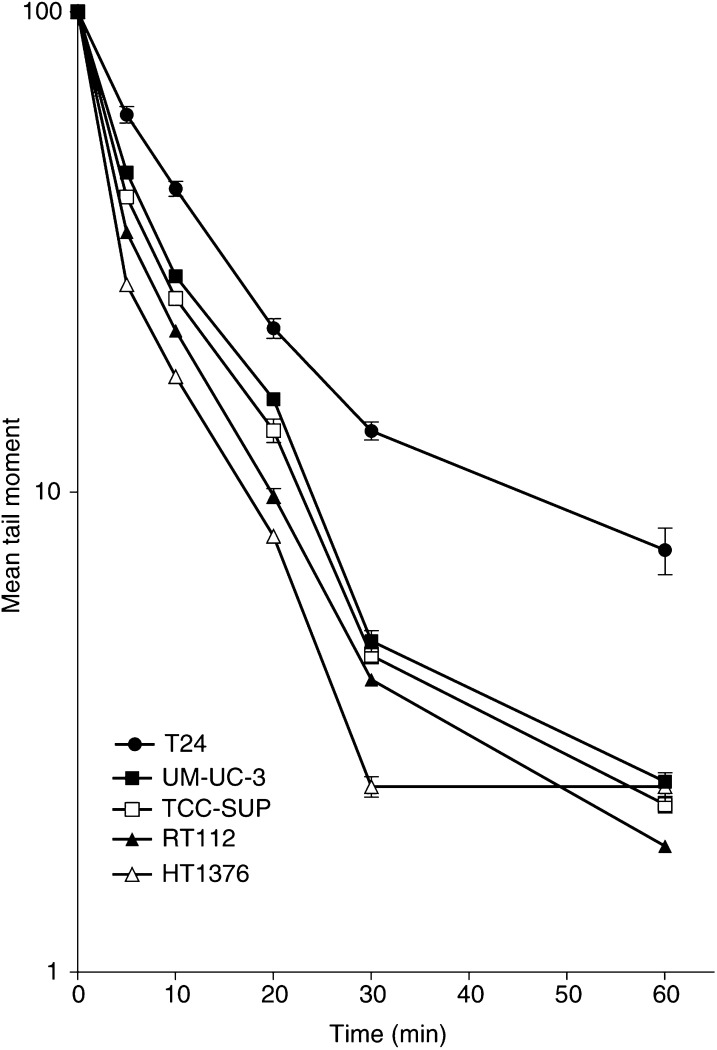
DNA repair in T24, UM-UC-3, TCC-SUP, RT112 and HT1376 cells after exposure to 4 Gy X-rays. Data points represent the mean±s.e. from three independent experiments. Error bars are shown only when they exceed the size of the symbol.

Repair of DNA damage was examined by incubating the cells at 37°C for up to 1 h after exposure to 4 Gy X-rays (Figure 5

). The time taken to repair half the initial DNA damage (Table 3

**Table 3:** Correlation coefficients obtained when SF2 is plotted against initial DNA damage (0 min), and remaining damage at a range of repair times (5–30 min)

**Repair time**	**Correlation coefficient (*r*^2^)^b^**
0 min	0.974
5 min	0.976
10 min	0.967
20 min	0.938
30 min	0.993
*T*_1/2_^c^	0.769

a SF2=the fraction of clonogenic cells surviving following 2 Gy X-irradiation.

b Correlation coefficient calculated from plots of SF2 against DNA damage 0–30 min following X-irradiation at 4 Gy. that is, residual − control damage/initial−control damage at time points 5–30 min for each cell line, except T24. A linear curve fit was applied and the correlation coefficient calculated.

c *T*_1/2_ =time point when half the initial damage (adjusted for background level) has been repaired. Residual DNA damage calculated by subtracting the control tail moment from that remaining at each time point in Fig. 5.

) showed a poorer correlation with SF2 (*r*^2^=0.769). By 45–60 min, repair was either complete or occurring at a much slower rate than initially. The extent of repair/residual damage at all time points (5, 10, 20 and 30 min) correlates well with SF_2_ (Tables 2 and 3); the correlation is most significant at 30 min *r*^2^=0.99 (Table 3, Figure 6

**Figure 6:**
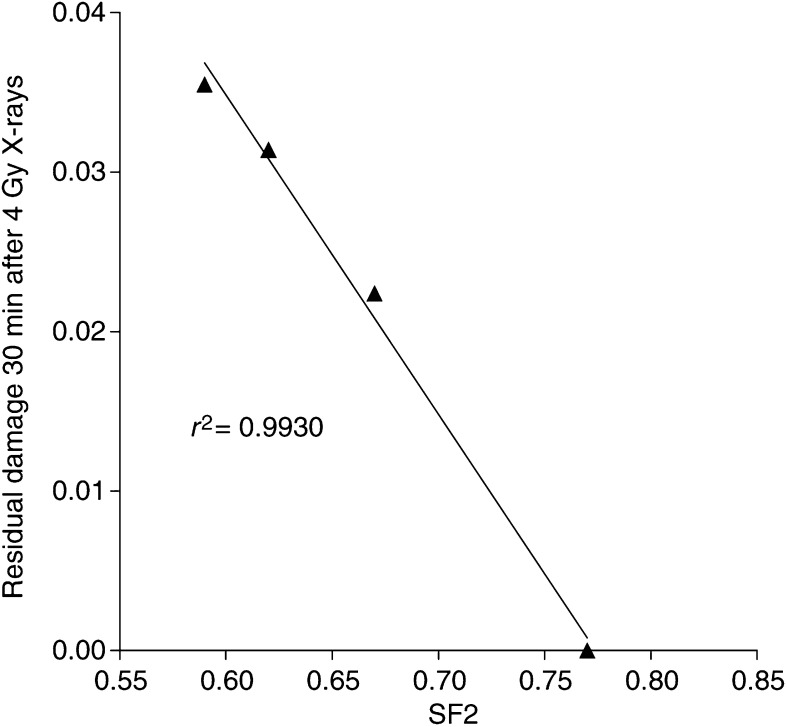
Correlation between SF2 and residual damage 30 min/initial damage following 4 Gy X-rays for the UM-UC-3, TCC-SUP, RT112 and HT1376 cell lines. Error bars are not shown on these graphs; they are included in Table 2.

).

## DISCUSSION

The ability to predict the radiosensitivity of individual tumours has long been the ‘holy grail’ of radiation biology (Lehnert, 2000). In this paper, we present data showing that the ACA can be used as a surrogate measure in the prediction of tumour cell radiosensitivity; the results are supported by two further papers (Dunne *et al*, 2003; Moneef *et al*, 2003).

Prior to using the ACA, we attempted to measure radiosensitivity in 24 primary bladder tumours using the soft agar clonogenic assay of West *et al* (1997). However, the success rate was very low (8%), this was attributed to the problem of achieving prolonged cell growth in this assay. The ACA offers an alternative method for detecting radiosensitivity, and is attractive as a potential clinical test as it requires a small number of cells and results can be available within a few hours (Fairbairn *et al*, 1995).

In many studies using the ACA cells have been irradiated in Eppendorf tubes prior to analysis. We used this method in a preliminary study (McKelvey-Martin *et al*, 1998) when well-defined radiation dose-response curves were observed with the greatest initial DNA damage displayed by the radiosensitive cell line (UM-UC-3) and the least by the radioresistant cell line (HT1376). However, it was difficult to determine the extent of DNA damage at low, clinically relevant, radiation doses (1–2 Gy). We have increased the sensitivity of the assay by embedding the cells in agarose prior to irradiation at 0°C; slides can then be placed in ice-cold lysis solution immediately following irradiation. Since radiation-induced DNA damage is repaired very rapidly (*t*_1/2_∼2–5 min; Table 2), it is inevitable that some repair will occur if cells are embedded in warm agarose after irradiation (Figure 2). Even if preembedded cells are irradiated (0–6 Gy) at room temperature, as compared to on ice, a smaller tail moment is obtained (our unpublished data); this supports the contention that every effort must be made to minimise repair during radiation exposure and up to the time of lysis. It is therefore critical that cells are preembedded and cooled on ice for any study using the ACA to measure initial radiation-induced DNA damage. With this modification, cell lines of different SF2 values could be separated using doses above 1.0 Gy. Recently, it has been shown that the half-life of repair of human head and neck tumours is about 4 min, showing that the rate of DNA repair following irradiation of human tumours *in situ* is of a similar order of magnitude to that *in vitro* (Terris *et al*, 2002).

Since the main aim of this study is to find a surrogate for the overall biological response in an individual tumour, the composite of factors that contribute to this cellular radiosensitivity should be reflected by the response measured. Of particular importance is our observation that the tail moment measured immediately after radiation exposure correlated with radiation dose (Figure 3) and can predict for cell survival in the clinically relevant range (Figure 4; *r*^2^=0.9514). Even the T24 cell line, which has a significantly more pronounced shoulder on the cell survival curve, shows good agreement between the two measures. It can be seen from the studies summarised in Table 1 that there has been surprisingly few similar studies of tumour cells using the ACA. Olive *et al* (1990) used the ACA to compare the radiosensitivity of infiltrating macrophages with tumour cells in a mouse tumour model; no differences in the cell populations was observed. Our initial study (McKelvey-Martin *et al*, 1998) did show correlation (see above) and this is supported by a study in three ovarian tumour cell lines (Bacova *et al*, 2000). Two studies do not support our findings, however they irradiated the cells prior to embedding thus reducing the sensitivity of their procedure (Muller *et al*, 1994; Bergqvist *et al*, 1998). In addition, our study has been confirmed by the concurrently presented studies of bladder tumour cells (Moneef *et al*, 2003) and colorectal tumour cells (Dunne *et al*, 2003) suggesting that the method is robust and reproducible in different tumour types and different laboratories. Indeed, it may be that our version of the ACA is capable of exposing differences that are masked in other variants of the same assay.

In contrast to the paucity of studies on SSBs there have been many studies using a range of assays to measure DSBs. The rationale for these investigations has been the general agreement that DSBs are the most critical of DNA-damaging lesions (Ward, 1988). However, the studies have shown little agreement as to whether initial DSBs vary between cell types. For example, in one review of 29 studies of neutral filter elution and neutral gel electrophoresis no consensus was found as to the correlation of survival with initial DSBs and rejoining (Olive *et al*, 1994). In a comparison of radiosensitive and radioresistant tumour cells using PFGE, the ACA and the halo assay, only the halo assay showed differences in initial damage. It was suggested, however, that the presentation of the DNA damage in the three assays might explain the differences (Woudstra *et al*, 1996). Chromatin structure, and its response to assay conditions, have also been implicated in responses observed with the halo DNA damage assay (Malyapa *et al*, 1994,^1996^). A major drawback of the NCA, and other methods carried out under neutral conditions, is the lack of sensitivity of these assays, necessitating the measurement of DSBs after exposure of cells to high radiation doses (>10 Gy) that will essentially kill all the cells. However, a number of factors can influence cell survival following radiation exposure, and the studies of Malaise *et al* (1987) and Deacon *et al* (1984) suggest that for a predictive test of radiosensitivity the measurements must be made at doses <6 Gy. Therefore studies of DSBs, although useful in understanding physico-chemical effects of radiation on DNA, have less utility in defining clinically relevant parameters. Indeed, it could be argued that assays of SSBs can be used as a surrogate for DSBs although direct evidence of this assumption is inferred rather than proven; several authors quote an implied linear ratio of DSBs to SSBs, for example, Olive and Johnston (1997). However, we propose that this assumption may be a better basis for evaluation of cellular radiosensitivity at low doses (2–4 Gy) and it may ultimately provide a predictive test that can be translated to the clinic, we are currently investigating this.

The literature on the correlation of DNA damage and clonogenicity is confusing. Differences in initial damage appear to depend primarily on both the assay and the specific conditions used in a particular laboratory. Many studies have used cells of different species and tissue origins and attempted to make direct comparison of radioresponsiveness; this may also result in misleading conclusions. For example, studies on fibroblasts tend to show a more limited, if any, difference in initial damage, a finding with which we would concur (our unpublished data). There is currently limited data available to explain the reason(s) for this variation and we can only speculate that differences in chromatin structure, antioxidant and thiol levels may all have a role to play (Kapiszewska *et al*, 1992; Woudstra *et al*, 1996; Mateos *et al*, 1998; McMillan *et al*, 2001). One group have suggested that initial damage, as measured in the halo assay and ACA, may not reflect overt strand breaks, but a loosening of chromatin structure which may vary between cell lines (Malyapa *et al*, 1994,^1996^). In a recent study (Rajab, 2002), it has been shown that nuclear texture, which may reflect differences in chromatin structure/organization, also correlates with radiosensitivity in bladder cancer cells. Whatever the explanation, it seems that some cells clearly have more disruption to their chromatin when irradiated than others. This seems to put a greater strain on the cell's repair capacity since, although the repair rates vary less, the time to return to background levels is longer for cells that have greater initial damage (Table 2; Figure 5; see also Moneef *et al* (2003).

When the ability to repair DNA damage following X-irradiation was compared to SF2, the cell lines maintained the same rank order as in the clonogenic assay (Figure 5). Although the time to repair half the initial damage was slower in radiosensitive cells, the differences were less significant (*r*^2^=0.769). The damage remaining at 30 min compared to SF2 also correlated very well for all cell lines (*r*^2^=0.993; Figure 6). Again repair and radiosensitivity have been found to correlate in some studies but not others. For example Daza *et al* (1997) have shown that although initial SSBs varied with radiosensitivity, initial DSBs and repair did not (using PFGE). Initial damage therefore appears to have a greater influence on a cell's overall response to radiation, and there appears to be some limitation in the capacity of the cells to repair at the initially rapid repair rate. This leaves those cell types with greater initial damage also with a greater residual damage as the second, slower repair phase, becomes dominant. As a consequence, the radiosensitive cells are left with ‘residual’ DNA damage for a much longer period than the resistant cells. It should be noted that T24 cells were excluded from the correlations of repair since they clearly change their radioresponsiveness between 2 and 4 Gy. On reflection, it might have been better to carry out the repair study at 2 Gy; this was not carried out because the differences in tail moment between the cell lines, although significant, were not large enough to follow repair for more than a limited time period. However, it now seems clear that initial damage is the most useful parameter and provides the best (and incidentally the easiest) surrogate measure of radiosensitivity.

Initially, the T24 cell line had a shallow survival curve, however, there was a much greater rate of change in radiosensitivity between 2.0 and 4.0 Gy making comparison of the SF_2_ with repair at 4 Gy problematic. However, this ‘problem’ can also be seen as a potential advantage. The change from resistant to sensitive phenotype in both assays, supports our contention that one assay predicts for the other. In addition, the influence of the position and change in the shoulder of the survival curve underlines the importance of carrying out predictive tests at clinically relevant doses. Clearly, for some tumours the wrong conclusion could be arrived at if radiosensitivity is predicted using a dose, even a few Gy, above that used in the clinic. This conclusion also concurs with seminal studies of Malaise *et al* (1987) and Deacon *et al* (1984), updated by Steel (1996) in which they provide considerable evidence that the radiosensitivity of tumours is most reliably measured at 2 Gy. The clinical studies of West and colleagues would support this proposition (West *et al*, 1997; Bjork-Eriksson *et al*, 2000).

In conclusion, although many studies have used the comet assay to measure radiation induced DNA damage and repair, there are only very few that have used ACA to compare several tumour cell lines of the same tissue origin. Studies on tumour cells, lymphocytes and fibroblasts are rather contradictory and this may indicate that there are differences in the DNA conformation and sensitivity to radiation in cells of different origins. We have attempted to avoid this problem by restricting ourselves to bladder tumour cells; a second parallel study, using our method and restricted also to bladder tumour cells, confirms our findings (Moneef *et al*, 2003), as does a study of colorectal tumours (Dunne *et al*, 2003). Although a complete understanding of the factors underlying the responses measured eludes us the repetition of this study in two other situations/laboratories suggests that, with our method, we can measure differences in cellular responses to radiation that can predict for tumour cell survival in a clinically relevant dose range. We consider that the evidence is now sufficient to compare the radiosensitivity of biopsy samples from human bladder tumours using initial damage at 2 Gy as the simplest and most reliable measure. A preliminary clinical trial is currently being carried out by Dr Jones and colleagues in Leicester, UK.
